# The effects of BDNF rs6265 and FGF21 rs11665896 polymorphisms on alcohol use disorder-related impulsivity in Han Chinese adults

**DOI:** 10.3389/fpsyt.2024.1339558

**Published:** 2024-04-24

**Authors:** Shizhuo Yang, Fan Wang, Lanrong Sun, Xinqian Liu, Siyuan Li, Yingjie Chen, Lingling Chen, Zeheng Pan, Yimin Kang, Yu-Hsin Chen, Wei Wang, Li Chen, Xiaokun Li, Chonghui Tang, Yanlong Liu

**Affiliations:** ^1^ Department of Neurosurgery, Affiliated Cixi Hospital, Wenzhou Medical University, Ningbo, China; ^2^ School of Pharmaceutical Science, Wenzhou Medical University, Wenzhou, China; ^3^ Beijing Hui-Long-Guan Hospital, Peking University, Beijing, China; ^4^ School of Mental Health, Wenzhou Medical University, Wenzhou, China; ^5^ Cixi Biomedical Research Institute, Wenzhou Medical University, Ningbo, China; ^6^ Psychosomatic Medicine Research Division, Inner Mongolia Medical University, Hohhot, China; ^7^ Zhejiang Provincial Clinical Research Center for Mental Disorders, the Affiliated Wenzhou Kangning Hospital, Wenzhou Medical University, Wenzhou, China

**Keywords:** alcohol use disorder, alcohol withdrawal, impulsivity, BDNF, FGF21

## Abstract

**Introduction:**

Patients with alcohol use disorder (AUD) often experience repeated withdrawal. Impulsivity is the most relevant factor influencing successful withdrawal. Brain-derived neurotrophic factor (BNDF) and fibroblast growth factor 21 (FGF21) are associated with impulsivity. Previous studies on the differential effects of BDNF or FGF21 on impulsivity have focused on single-gene effects and have inconsistent results. We aim to investigate the effects of BDNF rs6265 and FGF21 rs11665896, individually and together, on impulsivity during alcohol withdrawal in patients with AUD.

**Methods:**

We recruited 482 adult Han Chinese males with AUD and assessed their impulsivity using the Barratt Impulsivity Scale. Genomic DNA was extracted and genotyped from peripheral blood samples. Statistical analysis was conducted on the data.

**Results:**

The T-test and 2 × 2 analysis of variance were used to investigate the effects of the genes on impulsivity. There was a significant BDNF × FGF21 interaction on no-planning impulsiveness (*F* = 9.15, *p* = 0.003, *η^2^p* = 0.03). Simple main effects analyses and planned comparisons showed that BDNF rs6265 A allele × FGF21 rs11665896 T allele was associated with higher no-planning impulsiveness. Finally, hierarchical regression analyses revealed that only the interaction of BDNF and FGF21 accounted for a significant portion of the variance in no-planning impulsiveness.

**Conclusion and significance:**

The combination of BDNF rs6265 A allele and FGF21 rs11665896 T allele may increase impulsivity and discourage alcohol withdrawal. Our study provides a possible genetic explanation for the effects of associated impulsivity in patients with AUD from the perspective of gene-gene interactions.

## 1 Introduction

Alcohol is the most widely used addictive substance in the world, and China’s alcohol market has become one of the world’s largest (1). People with harmful drinking patterns often suffer from alcohol use disorder (AUD) that impairs health and functioning (2). AUD is a global health problem ranking seventh among the leading causes of death (3). When long-term drinkers suddenly stop drinking, AUD can cause alcohol withdrawal syndrome, which is an understudied subtype of AUD (4).

Alcohol withdrawal syndrome can lead to impulsivity, which is a constituent of human behavior in which individuals assume appropriate risks and pursue novel opportunities (5, 6). Impulsivity is associated with many at-risk behaviors, including suicidality, substance abuse, and criminal actions (7, 8). It is essential to identify subjects with high impulsivity tendencies, which can help alcohol-dependent patients successfully quit drinking quickly and reduce the risk of harmful behaviors. Not all individuals experience impulsivity in the context of alcohol withdrawal (9). Studies showed that impulsive behavior is associated with genetics linking individual differences to specific allelic variants arising from single-nucleotide polymorphisms (10, 11). Several genes are associated with impulsivity and aggression in alcohol-dependent individuals, including brain-derived neurotrophic factor (BDNF) (12), FGF21 (13), tryptophan hydroxylase type 2 (14), 5-HT receptor 2A (15), and catechol-O-methyl transferase (16).

BDNF is the most prevalent growth factor in the central nervous system. BDNF participates in developing the central nervous system and neuronal plasticity (17). The most common BDNF single-nucleotide polymorphism (SNP) in humans is at codon 66 (rs 6265), which can lead to a val-to-met (V66M) protein variant (18). Studies showed that BDNF is associated with impulsivity, and can be a biological marker for impulsive behavior (19). Findings regarding the BDNF rs6265 Met allele and impulsivity have been inconsistent. Bergman and Su et al. found that the BDNF rs6265 Met allele was associated with higher impulsivity in children with attention-deficit hyperactivity disorder and methamphetamine abusers (20, 21). In contrast, Boscutti et al. found that the BDNF rs6265 Met/Met genotype was associated with reduced impulsivity levels (22).

Fibroblast growth factor 21 (FGF21) is a member of the FGF19 superfamily (23). It mediates its biological effects by binding to a co-receptor, β-klotho (24). FGF21 can cross the blood-brain barrier (25) and is significantly associated with alcohol craving (26). FGF21 can modulate the functions of hypothalamic-pituitary-adrenal axis, which is associated with suicide and impulsive aggression (27, 28). A study found that a decrease in FGF21 level associated with serotonin and dopamine in the cerebrospinal fluid leads to higher impulsivity (29); the transcription level of FGF21 was influenced by miRNA. FGF21 rs11665896 is located at the 3′UTR region, where target sites for miRNAs are located, and the change of G for T resulting from this SNP could affect miRNA binding, reducing FGF21 transcription (30).

Studies found that BDNF and FGF21 are associated with alcohol dependence and impulsivity (31, 32). In addition, the link between BDNF SNP and AUD has been reported in some critical literature. An epidemiological study of 377 Japanese male alcoholics reported that, for the G196A genotype, people with the A allele develop alcohol abuse earlier than those who do not (33). Regarding the effect of Val66Met polymorphism of the BDNF gene on AUD, epidemiological studies have shown that BDNF gene Val66Met polymorphism increases vulnerability to alcohol dependence (34). However, another animal study suggests that BDNF Val66Met polymorphism dose not play a significant role in the genetic predisposition to alcohol dependence or violent tendencies (35). The association of BDNF and FGF21 genes’ variations and interactions with AUD-related impulsivity needs further investigation. Therefore, this study investigated the effects of two-gene variants, BDNF rs6265 and FGF21 rs11665896, individually and in combination, on impulsivity in AUD patients during alcohol withdrawal.

## 2 Materials and methods

### 2.1 Participants

We recruited 482 participants from several hospitals in northern China. All participants are Han Chinese men hospitalized for alcohol use disorders. The inclusion criteria were diagnosis of alcohol dependence by at least two trained psychiatrists according to the DSM-IV and sufficient literacy skills. The exclusion criteria were 1) a history of substance abuse or dependence other than nicotine, 2) participants or their first-degree relatives with a history of severe psychiatric or neurological disorders, and 3) cardiovascular, liver, or kidney disease.

All AUD patients voluntarily or passively came to the hospital on the day of drinking or the day after their last drinking session due to mental and behavioral disorders caused by alcohol consumption. Then, they experienced three weeks of abstinence in the hospital. After that, the participants were asked to complete questionnaires and provide a blood sample for DNA extraction. All patients provided written informed consent and were told the blood sample would be subjected to a gene assay. The institutional review board of the Inner Mongolian Medical University approved the study. All procedures performed in this study involving human participants followed the Helsinki Declaration.

### 2.2 Impulsivity

Impulsivity was assessed using the Barratt Impulsiveness Scale (BIS-11) Chinese Version. The BIS contains 30 items rated on a five-point Likert scale ranging from “no” to “always.” The BIS evaluates impulsivity in three domains: no-planning, motor, and cognitive impulsiveness (36). A higher score on the BIS indicates a higher level of impulsivity. The BIS has high internal consistency with a Cronbach’s α of 0.80 (37).

### 2.3 Genotyping

Genomic DNA was extracted from each participant 5 mL of peripheral blood using standard techniques. The BDNF rs6265 and FGF21 rs11665896 SNPs were genotyped using 5’ nuclease fluorescent TaqManTM primers (Applied Biosystems, Foster City, CA). Reactions were performed according to the manufacturer’s protocol. All laboratory procedures were carried out in a manner blind to case-control status. The conditions of polymerase chain reaction were as follows: 50°C for 2 min, 95°C for 10 min, followed by 50 cycles of 95°C for 15 s and 60°C for 1 min. BDNF rs6265 was genotyped with primers: (forward) 5’ GGACTCTGGAGAGGTGAAT-3’ and (reverse) 5’ CTCATCAGCTCTTCTATC -3’. FGF21 rs11665896 was genotyped with primers: (forward) 5’ TGTGTGGTGTCTGAGGGAAG-3’ and (reverse) 5’ GAAGTCAAGAGATGGAGAGCA-3’. Ten percent of the DNA samples were duplicated randomly and tested, and no-fault genotyping was found.

For the BDNF gene, there were 125 GG, 108 AA and 249 AG carriers. We grouped AA with AG to form an “A allele” (n = 355), with the remaining carriers categorized as “GG homozygote” (n = 125) genotype. For the FGF21 gene, there were 233 GG, 52 TT and 197 GT carriers. We grouped TT with GT to form a “T allele” (n = 249) with the remaining carriers categorized as “GG homozygote” (n = 233) genotype.

### 2.4 Statistical analyses

Pearson correlations were examined between marital status, living status, age, educational years, no-planning, cognitive, and motor impulsiveness. Then, the Hardy-Weinberg equilibrium for genotype distributions of BDNF rs6265 and FGF21 rs11665896 was tested using the c2 test for goodness of fit. To investigate the effects of genes on impulsivity, we conducted independent t-tests for each genotype and a 2 × 2 analysis of variance to examine BDNF rs6265 × FGF21 rs11665896 interaction on individual dimensions of impulsivity. Significant interactions were explored using simple main effects analyses and planned comparisons. Finally, we conducted hierarchical regression analyses to determine the specific and interactional effects of BDNF rs6265 × FGF21 rs11665896 on no-planning impulsiveness.

## 3 Results

### 3.1 Descriptive statistics

We included 482 participants. The mean age was 41.42 ± 10.24 years. The average number of education years was 11.18 ± 2.81 years. Most participants were married (71.6%) and living with family (78.4%). The impulsivity scores were no-planning impulsiveness (41.54 ± 19.30), cognitive impulsiveness (40.47 ± 17.47), and motor impulsiveness (33.60 ± 18.06). Correlation analyses revealed that general demographic, age and educational years were significantly correlated with impulsivity (**Table 1**). Age was significantly positively associated with impulsivity (|*r*|s ≥ 0.12, *p*s < 0.01), and educational years were significantly negatively associated with impulsivity (|*r*|s ≥ 0.21, *p*s < 0.001). Marital status and living status showed no significant correlations with impulsivity.

**Table 1:** Descriptive statistics and correlations among study variables.

	1.	2.	3.	4.	5.	6.	7.
1.Marital status	1						
2.Living status	0.33^***^	1					
3.Age	0.30^***^	0.16^***^	1				
4.Educational years	-0.15^**^	-0.10^*^	-0.47^***^	1			
5.No-planning Impulsiveness	-.003	0.05	0.13^**^	-0.22^***^	1		
6.Cognitive Impulsiveness	0.04	0.08	0.12^**^	-0.22^***^	0.77^***^	1	
7.Motor Impulsiveness	0.05	0.05	0.12^**^	-0.21^***^	0.33^***^	0.26^***^	1
*M*	(–)	(–)	41.42	11.18	41.54	40.47	33.60
*SD*	(–)	(–)	10.24	2.81	19.30	17.47	18.06

^*^p < 0.05; ^**^p < 0.01; ^***^p < 0.001.

### 3.2 Effect of BDNF rs6265 and FGF21 rs11665896 genotypes on impulsivity

Before performing a single-gene effect test, Hardy-Weinberg equilibrium was established for each gene (**Table 2**). BDNF rs6265 and FGF21 rs11665896 were in Hardy-Weinberg equilibrium (*χ* ^2^ = 0.573, *p* = 0.449; *χ* ^2^ = 1.128, *p* = 0.288). The results of single-gene effect showed that there were no significant differences of impulsivity between BDNF rs6265 A allele carriers and GG homozygote carriers (*ts* < 1.56, *p* > 0.10), and there were no significant differences of impulsivity between FGF21 rs11665896 T allele carriers and GG homozygote carriers (*ts* < 0.95, *p* > 0.19) (**Table 3**). There were no significant single-gene effects of BDNF rs6265 or FGF21 rs11665896 on AUD-related impulsivity.

**Table 2:** Hardy-Weinberg equilibrium of BDNF rs6265 and FGF21 rs11665896.

	BDNF rs6265			FGF21 rs11665896		
Genotype	AA	AG	GG	GG	GT	TT
Number of people	108	249	125	233	197	52
Percentage	22.4%	51.7%	25.9%	48.3%	40.9%	10.8%
*χ^2^*	0.573			1.128		
*p*	0.449			0.288		

**Table 3:** Impulsivity scores for single-gene genotypes.

	BDNF rs6265			FGF21 rs11665896		
	BDNF: A allele (n =357)	BDNF: GG homozygote (n = 125)	*t*	FGF21: GG homozygote (n = 233)	FGF21: T allele (n = 249)	*t*
No-planning Impulsiveness	41.87 ± 19.64	40.60 ± 18.32	-0.63	41.75 ± 18.88	41.35 ± 19.71	0.82
Cognitive Impulsiveness	41.20 ± 18.04	38.38 ± 15.61	-1.56	40.42 ± 17.68	40.52 ± 17.31	0.95
Motor Impulsiveness	33.78 ± 18.26	33.10 ± 17.55	-0.36	32.50 ± 17.72	34.64 ± 18.39	0.19

### 3.3 Effect of the interaction of BDNF rs6265 and FGF21 rs11665896 genotypes on impulsivity

**Figure 1:**
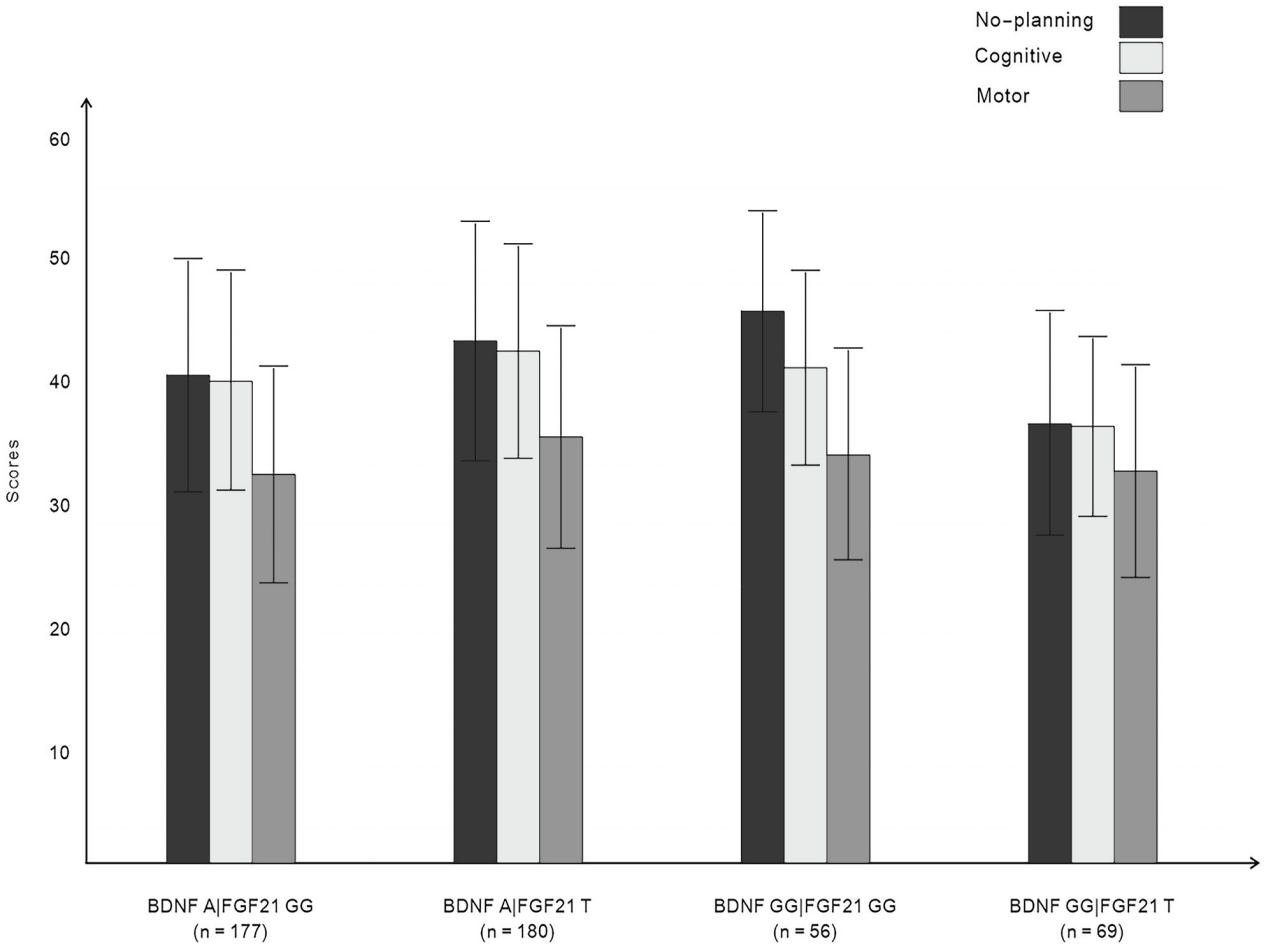
Impulsivity scores for BDNF rs6265 and FGF21 rs11665896.

**Figure 1** displays the impulsivity scores for the four allelic groups. BDNF rs6265 A allele and FGF21 rs11665896 T allele carriers showed higher levels of impulsivity. BDNF rs6265 GG homozygote and FGF21 rs11665896 T allele carriers showed lower levels of impulsivity. We subjected these impulsivity scores to analysis of variance with two between-subjects factors of BDNF rs6265 (A allele/GG homozygote) and FGF21 rs11665896 (T allele/GG homozygote) (**Table 4**). There was a significant BDNF × FGF21 interaction on no-planning impulsiveness (*F* = 9.15, *p* = 0.003, *η* ^2^ *p* = 0.03). However, this interaction effect was absent on cognitive impulsiveness (*F* = 3.55, *p* = 0.060, *η* ^2^ *p* = 0.01) and motor impulsiveness (*F* = 1.57, *p* = 0.21, *η* ^2^ *p* = 0.003).

**Table 4:** The interaction effects between BDNF and FGF21 polymorphisms on impulsiveness.

Parameter	Factor	*SS*	*df*	*MS*	*F*	*p*	*η* ^2^ *p*
No-planning Impulsiveness	BDNF	54.76	1	54.76	0.149	0.700	0.000
	FGF21	997.08	1	997.08	2.714	0.100	0.006
	Interaction	3362.47	1	3362.47	9.154	0.003	0.03
	Residual	175585.37	478	367.33			
Cognitive Impulsiveness	BDNF	603.12	1	603.12	1.99	0.159	0.004
	FGF21	194.03	1	194.03	0.64	0.424	0.001
	Interaction	1075.57	1	1075.57	3.55	0.060	0.01
	Residual	144967.01	478	303.28			
Motor Impulsiveness	BDNF	32.80	1	32.80	0.101	0.751	0.000
	FGF21	95.81	1	95.81	0.294	0.588	0.000
	Interaction	511.79	1	511.79	1.570	0.211	0.003
	Residual	155786.21	478	325.91			

Simple main effects analysis (**Table 5**) and planned comparisons (**Table 6**) were conducted to explore this significant interaction. There was a significant BDNF effect in FGF21 T allele carriers (*F* = 6.32, *p* = 0.01) but not in FGF21 GG homozygote carriers (*F* = 3.32, *p* = 0.07). There was a significant FGF21 effect in BDNF A allele carriers (*F* = 7.35, *p* = 0.007) but not in BDNF GG homozygote carriers (*F* = 1.85, *p* = 0.18). The planned comparisons showed higher no-planning impulsiveness for the BDNF rs6265 A allele than for BDNF rs6265 GG homozygote carriers (*t* = -2.51, *p* = 0.01) in the FGF21 T allele group and higher no-planning impulsiveness for FGF21 rs11665896 T allele relative to FGF21 rs11665896 GG homozygote carriers (*t* = -2.71, *p* = 0.007) in the BDNF A allele group. This significant difference was also absent in the FGF21 GG homozygote group (*t* = 1.80, *p* = 0.07) and the BDNF GG homozygote group (*t* = 1.36, *p* = 0.18).

**Table 5:** Sample main effect.

Effect	Fixed	*F*	*p*
BDNF	FGF21 GG	3.23	0.07
BDNF	FGF21 T	6.32	0.01
FGF21	BDNF GG	1.85	0.18
FGF21	BDNF A	7.35	0.007

**Table 6:** Planned comparisons.

Fixed	Contrast	Diff (*se*)	*t*	*p*
FGF21 GG	BDNF GG – BDNF A	5.28 (2.94)	1.80	0.07
FGF21 T	BDNF GG – BDNF A	-6.82 (2.71)	-2.51	0.01
BDNF GG	FGF21 GG – FGF21 T	2.76 (2.03)	1.36	0.18
BDNF A	FGF21 GG – FGF21 T	-9.35 (3.45)	-2.71	0.007

Finally, multivariate regression analysis was performed for the primary and interaction effects of BDNF and FGF21 on impulsiveness. BDNF rs6265 and FGF21 rs11665896 were entered as predictors of impulsiveness. Only the interaction of BDNF and FGF21 accounted for a significant portion of the variance in no-planning impulsiveness (*β* = -0.14, *t* = 2.195, *p* = 0.029). This significant interaction was absent for cognitive impulsiveness and motor impulsiveness.

## 4 Discussion

We investigated the effects of BDNF rs6265 and FGF21 rs11665896 on impulsivity with alcohol dependence during withdrawal. Previous studies showed that BDNF played a significant role in the processes of alcohol withdrawal syndrome (31), and FGF21 is related to AUD (32). The present study found no significant single-gene effect of BDNF rs6265 or FGF21 rs11665896 on AUD-related impulsivity. However, in the interaction analysis with two genes, we found that the interaction of BDNF rs6265 × FGF21 rs11665896 had a significant effect on no-planning impulsiveness in participants with AUD. When the BDNF rs6265 A allele was combined with the FGF21 rs11665896 T allele, no-planning impulsiveness was higher in patients with AUD.

BDNF rs6265 A allele and FGF21 rs11665896 T allele caused the production of mature protein (BNNF and FGF21) to decrease (30, 38). In a sample of individuals who had attempted suicide (39) and in attention-deficit hyperactivity disorder patients (40), low blood BDNF concentrations were associated with high levels of impulsivity. Low FGF21 levels in the cerebrospinal fluid led to impulsivity (29). Studies have shown that the higher the impulsivity, the higher the risk of being drink. Impulsivity can motivate alcohol consumption in a variety of ways. Stamates et al. found that individuals with high levels of impulsivity appear to have more significant negative internal and external motives for drinking (41). Moreover, high trait impulsivity, as measured with the BIS-11, may result in/from heavy drinking patterns in young social drinkers (42). In addition, impulsivity is related to a higher risk of relapse in alcohol use disorders (43, 44). FGF21 and BDNF have many similar biological effects. They have neuroprotective effects such as improving cognitive function and neurodegeneration (45–48). BDNF and FGF21 levels change after drinking alcohol (49, 50), suggesting there may be an interaction between BDNF and FGF21. Studies found that muscle-derived mediators such as FGF21 and cathepsin-B (CTSB) can pass through the blood-brain barrier and enhance neuroprotective markers including irisin and BDNF (51). Kang et al. found that FGF21 activated the AMPKα/SIRT1 signaling pathway, directly or indirectly inhibiting NF-κB expression and enhancing BDNF expression (45). Mice with the livers of BDNF mutants fed a high-fat diet contained abnormal levels of peroxisome proliferator-activated receptor and FGF21 transcripts (52).

In the present study, the BDNF rs6265 A allele or FGF21 rs11665896 T allele corresponded to lower BDNF and FGF21 protein levels. Decreases in FGF21 or BDNF may lead to a decrease in the other protein. The combination of the BDNF rs6265 A allele and the FGF21 rs11665896 T allele may correspond to the lowest BDNF and FGF21 levels in vivo.

Long-term heavy drinking can lead to inflammation and apoptosis (53, 54). Pro-BDNF and BDNF may produce opposite biological functions by signaling through p75NTR and TrkB, respectively (55). Anastasia et al. found that Met66proBDNF binds more effectively to the p75NTR/sortilin complex receptor than Val66proBDNF (56). Pro-BDNF-p75NTR activates the JNK pathway to upregulate p53 expression and initiate apoptosis (57). BDNF depletion is associated with neuroinflammation and neuronal apoptosis in Alzheimer’s disease (58). FGF21 relieved numerous inflammation-related metabolic disorders, including metabolic syndrome and cardiovascular diseases (59). Lu et al. found that FGF21 reduced senescence, apoptosis, and extracellular matrix degradation in osteoarthritis via the SIRT1-mTOR signaling pathway (60). These findings suggest that the effects of BDNF and FGF21 on impulsivity may be mediated by inflammation and apoptosis.

The mechanisms underlying higher impulsivity may also be associated with neurotransmitter changes, including serotonin and dopamine (61, 62). BDNF regulates mood, cognition, and response to stress and interacts with serotonergic, glutamatergic, cholinergic and dopaminergic neurotransmission (63, 64). Xu et al. found that the effect of CSF FGF21 on impulsivity may be related to the regulating effects of FGF21 on serotonin and dopamine in CSF (29). These findings suggest that BDNF and FGF21 may affect impulsivity by regulating neurotransmitters such as serotonin and dopamine.

The present study has some limitations. First, the measure of impulsivity used self-report scales, and data were collected using self-rating scales; therefore, reporting bias is unavoidable. Second, our study was cross-sectional and should be extended to longitudinal studies. Third, this study only included male patients. Nevertheless, some studies have found sex differences in drinking behavior, neural systems, and AUD treatment (65, 66). Schulte et al. found that while girls and boys may be facing similar vulnerabilities to problems with alcohol, boys begin to carry more risk as they move toward young adulthood (67). Agabio et al. found that women were older than men at the age of first drink, regular drinking, and onset of AUD, and progressed faster than men from regular use to AUD onset (68). More importantly, the sex difference occurs in BDNF SNP reported by alcohol-related studies. Female hBDNF^Val/Val^ mice exhibited a greater propensity toward stable ethanol self-administration than male mice of the same genotype in the operant paradigm (6). Male Met68BDNF mice exhibited a preference for alcohol over social interaction and were insensitive to the acute anxiolytic action of alcohol, which was driven by malfunction of BDNF in the ventral hippocampus of male mice (69). Therefore, further research in female patients with AUD is warranted in the future. Fourth, our study examined only one genotype of BDNF or FGF21 and should be investigated using gene set analysis. Finally, specific mechanisms should be explored using relevant molecular biomolecular methods. The strengths of this study include our more profound understanding of the impact of AUD-related impulsivity at the two-gene level.

## 5 Conclusion

In summary, our findings suggest that combinations of genotypes of BDNF and FGF21 have different effects on impulsivity during withdrawal in alcohol-dependent patients. The BDNF rs6265 A allele × FGF21 rs11665896 T allele is associated with higher no-planning impulsiveness. The combination of BDNF rs6265 A allele and FGF21 rs11665896 T allele maybe a risk to increase impulsivity, which is not conducive to quitting in alcohol-dependent patients. There is a lack of research on AUD based on genetic variations. Further study on genetic targets may improve understanding of AUD and provide treatment methods for AUD patients.

## Data availability statement

The raw data supporting the conclusions of this article will be made available by the authors upon reasonable request.

## Ethics statement

The studies involving humans were approved by the institutional review board of the Inner Mongolian Medical University. The studies were conducted in accordance with the local legislation and institutional requirements. The participants provided their written informed consent to participate in this study.

## Author contributions

SY: Data curation, Investigation, Validation, Writing – original draft. FW: Formal analysis, Resources, Writing – original draft. LS: Formal analysis, Software, Writing – original draft. XQL: Investigation, Writing – original draft. SL: Investigation, Writing – original draft. YC: Investigation, Writing – original draft. LLC: Investigation, Writing – original draft. ZP: Investigation, Writing – original draft. YK: Methodology, Project administration, Supervision, Writing – original draft. Y-HC: Methodology, Project administration, Supervision, Writing – original draft. WW: Methodology, Project administration, Supervision, Writing – original draft. LC: Methodology, Project administration, Supervision, Writing – original draft. XKL: Conceptualization, Data curation, Supervision, Writing – review & editing. CT: Conceptualization, Data curation, Supervision, Writing – review & editing. YL: Conceptualization, Project administration, Supervision, Writing – review & editing.
